# Monocyte subset distribution and surface expression of HLA-DR and CD14 in patients after cardiopulmonary resuscitation

**DOI:** 10.1038/s41598-021-91948-z

**Published:** 2021-06-11

**Authors:** Alexander Asmussen, Hans-Jörg Busch, Thomas Helbing, Xavier Bemtgen, Christian Smolka, Christoph Bode, Katrin Fink, Sebastian Grundmann

**Affiliations:** 1 Department of Cardiology and Angiology I, University Heart Center Freiburg - Bad Krozingen, Medical Center - University of Freiburg, Faculty of Medicine, University of Freiburg, Hugstetter Str. 55, 79106 Freiburg im Breisgau, Germany; 2 University Emergency Center, Medical Center - University of Freiburg, Faculty of Medicine, University of Freiburg, Sir-Hans-A.-Krebs-Straße, 79106 Freiburg im Breisgau, Germany; 3 Department of Cardiology, Heart Center Oldenburg, University of Oldenburg, Rahel-Straus-Str. 10, 26133 Oldenburg, Germany

**Keywords:** Acute inflammation, Diagnostic markers, Inflammatory diseases

## Abstract

Systemic inflammation is a major feature of the post-cardiac arrest syndrome. The three monocyte subpopulations are thought to play an important role in this inflammatory state because they are endowed with numerous pattern recognition receptors, such as CD14, that have been associated with ischemia–reperfusion injury. By contrast, an exaggerated antiinflammatory response has also been described following cardiac arrest, which may be mediated by downregulation of antigen presentation receptor HLA-DR. We report the composition of monocyte subpopulations and the expression of CD14 and HLA-DR following cardiac arrest. Blood specimens were collected from 32 patients at three timepoints in the first 48 h after cardiac arrest. Monocyte subset composition was determined by flow cytometry based on the expression of CD14, CD16, and HLA-DR. Monocyte subset composition and the expression of CD14 and HLA-DR were correlated with patient outcomes. The results were compared to 19 patients with coronary artery disease. Cardiac arrest patients showed a significant decline in the percentage of nonclassical monocytes. Monocyte CD14 expression was upregulated after 24 h and correlated with the time to return of spontaneous circulation. Downregulation of HLA-DR expression was observed mainly among classical monocytes and significantly correlated with the dose of norepinephrine used to treat shock. Downregulation of HLA-DR among nonclassical and intermediate monocytes was significantly associated with disease severity. Our data demonstrate the disturbance of monocyte subset composition with a significant decline in nonclassical monocytes at an early stage following cardiac arrest. Our findings suggest the simultaneous presence of hyperinflammation, as evidenced by upregulation of CD14, and monocyte deactivation, characterized by downregulation of HLA-DR. The extent of monocyte deactivation was significantly correlated with disease severity.

## Introduction

Systemic ischemia–reperfusion injury following cardiac arrest results in a unique pathophysiological condition known as the post-cardiac arrest syndrome (PCAS)^1^. PCAS comprises a tetrad of clinical components, namely (1) post-cardiac arrest brain injury, (2) post-cardiac arrest myocardial dysfunction, (3) systemic ischemia–reperfusion response, and (4) persistent precipitating pathology, which are held accountable for the poor prognosis of patients suffering cardiac arrest^1^.

Hyperinflammation is a hallmark of the “sepsis-like syndrome” observed during PCAS^2^. Systemic ischemia–reperfusion injury following cardiopulmonary resuscitation (CPR) leads to an increase in circulating pathogen-associated molecular patterns (PAMPs) and danger-associated molecular patterns (DAMPs), which serve as ligands for pattern recognition receptors (PRR)^2,3^. Upon activation, PRRs, such as Toll-like receptors (TLR) and their coreceptor CD14, have been shown to orchestrate sterile inflammation in response to cellular injury^4^. Monocytes are equipped with several PRRs and could thus possibly mediate systemic inflammation during PCAS, as previously described by our group^5^. As another feature of the “sepsis-like syndrome”, patients at the same time develop an immunosuppressive phenotype, which is characterized by monocyte hyporesponsiveness in vitro^2,5^. Along with the downregulation of antigen presentation receptor monocyte HLA-DR (mHLA-DR), monocyte hyporesponsiveness is considered a classical feature of immunoparalysis^6^. Expression analyses of CD14 and mHLA-DR were performed to further characterize the inflammatory response following cardiac arrest.

Human monocytes, which are subdivided into three subsets with distinctive inflammatory features based on the expression of CD14 and CD16, are considered key players in the context of hyperinflammatory disorders. CD14^++^CD16^−^ monocytes, which are referred to as “classical” monocytes, represent the predominant subset. CD16^+^ monocytes comprise two subsets, namely the CD14^++^CD16^+^ “intermediate” subset and the CD14^+^CD16^++^ “nonclassical” subset^7^. On a functional level, CD14^++^CD16^−^ monocytes act as scavenger cells and produce a broad range of antimicrobial agents^8,9^. Intermediate monocytes are potent inducers of inflammation^9,10^ and exert antigen presentation^8,9^. Nonclassical monocytes provide immune surveillance by patrolling the endothelium in a crawling behavior^11^. We hypothesized that PCAS would lead to a rearrangement in the composition of the monocyte population due to the specific characteristics of each subpopulation.

## Results

### Patient characteristics

Thirty-two patients who had suffered cardiac arrest (CPR group) and 19 patients with CAD were prospectively enrolled. Both groups showed a comparable prevalence of sex ratio, mean age, and past medical conditions (Table 1). The cardiovascular risk profile indicated a greater prevalence of dyslipidemia (CPR group 25% vs CAD group 68%; *p* = 0.003) and arterial hypertension (CPR group 47% vs CAD group 79%; *p* = 0.039) among the CAD group (Table 1).

**Table 1:** Baseline patient characteristics.

	CPR group (n = 32)	CAD group (n = 19)	*P* value
Age (years)	68.1 ± 10.4	68.9 ± 11.6	0.793
Sex (male:female)	25:7	15:4	1.000
**Medical history**			
Coronary artery disease	21 (66%)	19 (100%)	0.004
Peripheral artery disease	1 (3%)	1 (5%)	1.000
Chronic heart failure	5 (16%)	3 (16%)	1.000
Pulmonary hypertension	3 (9%)	0 (0%)	0.285
Chronic lung disease	6 (19%)	1 (5%)	0.236
Chronic liver disease	0 (0%)	0 (0%)	N/A
Chronic kidney disease	4 (13%)	5 (26%)	0.266
**Cardiovascular risk factors**			
Arterial hypertension	15 (47%)	15 (79%)	0.039
Diabetes	7 (22%)	6 (32%)	0.515
Dyslipidaemia	8 (25%)	13 (68%)	0.003
Smoking	12 (38%)	12 (63%)	0.091
Overweight	5 (16%)	7 (37%)	0.101
**Interventions**			
Coronary angiography < 12 h prior to study enrolment	27 (84%)	8 (42%)	0.004
PCI < 12 h prior to study enrolment	15 (47%)	8 (42%)	0.779

*CPR* cardiopulmonary resuscitation, *CAD* coronary artery disease, *PCI* percutaneous coronary intervention, *N/A* not applicable.

Of patients who had undergone CPR, 66% had significant CAD compared to 100% in the reference group (*p* = 0.004). Significantly more patients in the CPR group underwent coronary angiography prior to (< 12 h before) study enrollment (CPR group 84% vs CAD group 42%; *p* = 0.004), which, however, did not lead to a greater extent of coronary revascularization through percutaneous coronary intervention (CPR group 47% vs CAD group 42%; *p* = 0.779) (Table 1).

Most of the study population was resuscitated from out-of-hospital cardiac arrest (91%). Most patients had ventricular fibrillation or ventricular tachycardia as the initial rhythm presentation (62%). To a lesser extent, patients presented with asystole or pulseless electrical activity (38%). The mean duration from collapse to return of spontaneous circulation (ROSC) was 29 ± 20 min. All patients in the CPR group were comatose at the time of hospital admission and received targeted temperature management at 33 °C. The probability of an unfavorable neurological outcome (Cerebral Performance Category (CPC) score 3, 4, and 5) at the time of hospital discharge, as assessed by Cardiac Arrest Hospital Prognosis (CAHP) score and Out-of-Hospital Cardiac Arrest (OHCA) score, was 87.7% (176.6 points) and 66.3% (26.3 points), respectively. Finally, 77.4% of patients had an unfavorable neurological outcome after cardiac arrest. The 30-day mortality rate in the CPR group was 63% (Table 2).

**Table 2:** Characterization of patients after cardiopulmonary resuscitation.

	CPR group (n = 32)
**CPR scene**	
OHCA	29 (91%)
IHCA	3 (9%)
**Etiology of cardiac arrest**	
Cardiac	20 (62%)
Non-cardiac	7 (22%)
Unknown	5 (16%)
**Initial rhythm**	
VT/VF	20 (62%)
Asystole/PEA	12 (38%)
**Time from collapse to CPR (min)**	2.9 ± 4.2
**Time from collapse to ROSC (min)**	29.2 ± 19.8
**Sequential Organ Failure Assessment (SOFA) Score**	
Day 1 after ROSC	10.3 ± 1.8
Day 2 after ROSC	10.8 ± 1.6
Day 3 after ROSC	11.3 ± 1.6
**Simplified Acute Physiology Score II (SAPS II) at day 1**	80.9 ± 7.5
**Acute Physiology and Chronic Health Evaluation II Score (APACHE II) at day 1**	31.4 ± 4.0
**Cardiac Arrest Hospital Prognosis Score (CAHP) at time of admission**	176.6 ± 43.2
**Out-of-Hospital Cardiac Arrest Score (OHCA) at time of admission**	26.3 ± 14.7
**Serum lactate at time of admission (mM)**	7.5 ± 5
**Targeted temperature management (33 °C)**	32 (100%)
**Consecutive organ failure**	
Acute heart failure	10 (31%)
Acute respiratory failure	5 (16%)
Acute liver failure	0 (0%)
Acute renal failure	9 (28%)
**Cerebral performance category (CPC)**	
1, 2	7 (22.6%)
3, 4, and 5	24 (77.4%)
**30-day mortality rate**	20 (63%)

Acute heart failure was defined by clinical signs of cardiac decompensation or cardiogenic shock. Acute renal failure was defined as an increase in serum creatinine ≥ 0.3 mg/dl or an ≥ 1.5-fold increase from baseline creatinine within the first 48 h.

Acute liver failure was defined as an increase in total bilirubin serum levels and an increase in the International Normalized Ratio above the reference values of our central laboratory.

Acute respiratory failure was defined based on an oxygenation index (ratio of PaO_2_ (mmHg) and FiO_2_ (%)) ≤ 200 mmHg.

CAHP and OHCA score were only calculated in patients after out-of-hospital cardiac arrest. One patient was lost to follow-up after study enrollment.

*CPR* cardiopulmonary resuscitation, *OHCA* out-of-hospital cardiac arrest, *IHCA* in-hospital cardiac arrest, *VT* ventricular tachycardia, *VF* ventricular fibrillation, *PEA* pulseless electrical activity, *ROSC* return of spontaneous circulation.

Mean time from ROSC to blood sampling was 6.7 ± 3.2 h for the first (CPR t1), 25.3 ± 3.5 h for the second (CPR t2), and 48.2 ± 3.4 h for the third blood specimen (CPR t3). A summary of routine laboratory values is provided in Table 3.

**Table 3:** Laboratory tests.

	CPR group (n = 32)	CAD group (n = 19)	*P* value
**Laboratory tests**			
At admission:			
White blood count (10^3^/µl)	15.00 ± 6.76	7.53 ± 2.19	0.000^a^
Platelet count (10^3^/µl)	197.13 ± 66.03	233.58 ± 66.67	0.063^a^
C-reactive protein (mg/l)	10.24 ± 14.54	5.24 ± 4.05	0.453^a^
Procalcitonin (ng/ml)	2.06 ± 7.65		N/A
24 h after ROSC			
White blood count (10^3^/µl)	11.48 ± 4.82		0.076^b^
Platelet count (10^3^/µl)	183.14 ± 66.70		0.414^b^
C-reactive protein (mg/l)	38.50 ± 37.47		0.000^b^
Procalcitonin (ng/ml)	3.68 ± 7.57		0.000^b^
48 h after ROSC			
White blood count (10^3^/µl)	11.02 ± 4.23		0.050^b^
Platelet count (10^3^/µl)	163.54 ± 56.37		0.041^b^
C-reactive protein (mg/l)	108.36 ± 53.21		0.000^b^
Procalcitonin (ng/ml)	3.88 ± 6.18		0.000^b^

Shown are the patients’ inflammation blood tests at the time of admission, as well as 24 h and 48 h after ROSC.

*CPR* cardiopulmonary resuscitation, *CAD* coronary artery disease.

^a^CPR group at admission versus CAD group.

^b^CPR group versus CPR group at admission.

### Monocyte subset composition

**Figure 1:**
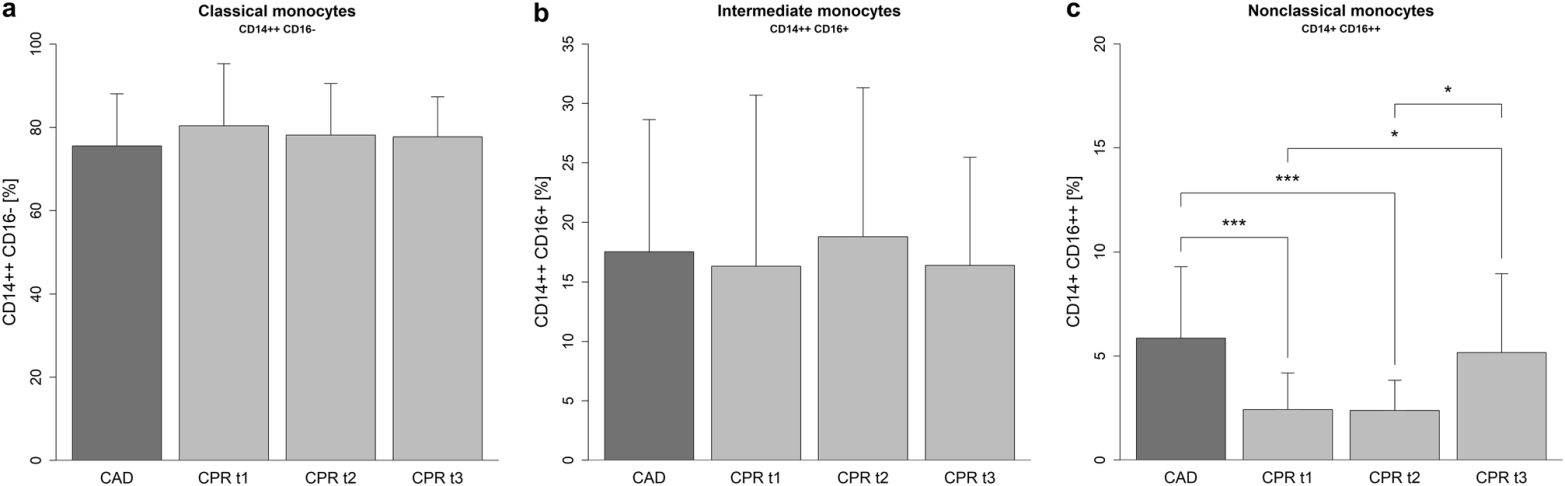
Monocyte subset composition in resuscitated patients and the control group. The relative fraction of monocyte subsets from the CPR group in the first 12 h (CPR t1: n = 22), after 24 h (CPR t2: n = 21) and 48 h (CPR t3: n = 16) following ROSC, and the control group (CAD: n = 19) is shown. Statistical hypothesis testing was performed using the Kruskal–Wallis test and post-hoc analysis with all-pairwise comparisons using the Dunn–Bonferroni approach (*: *p* ≤ 0.05; ***: *p* ≤ 0.001).

Changes in the relative composition of monocyte subsets were studied during the first 48 h of PCAS (Fig. 1). The calculated absolute count of monocyte subsets can be found in Supplementary Figure S1. Classical monocytes constituted the largest subset in both groups. The relative proportion of classical monocytes did not change during the study period (CAD 75.53 ± 12.53; CPR t1 80.41 ± 14.91; CPR t2 78.13 ± 12.47; CPR t3 77.73 ± 9.60 (%)). Intermediate monocytes made up the second largest subset, and their relative share of total monocytes likewise did not change over time (CAD 17.54 ± 11.10; CPR t1 16.33 ± 14.36; CPR t2 18.77 ± 12.54; CPR t3 16.41 ± 9.07 (%)). Interestingly, we observed a significant reduction in the proportion of nonclassical monocytes during the first 24 h of PCAS. The fraction of nonclassical monocytes returned to levels comparable to the control group on day 2 after CPR (CAD 5.86 ± 3.44; CPR t1 2.42 ± 1.76; CPR t2 2.38 ± 1.45; CPR t3 5.17 ± 3.80 (%)). Similar results were obtained in the analysis of the calculated absolute number of nonclassical monocytes following cardiac arrest. When only compared to the control group, a statistically significant decline in nonclassical monocytes could be observed in the first 12 h after ROSC with a trend to lower numbers after 24 h.

### Correlation of monocyte subset composition and clinical markers

We hypothesized that changes in the composition of monocyte subsets might correlate with severity of PCAS and patient outcomes. Therefore, correlation analyses were performed between the composition of monocyte subgroups and (1) 30-day mortality, neurological outcome, and both critical care (SOFA, SAPS II, APACHE II) and cardiac arrest specific (CAHP, OHCA) predictive scoring systems, (2) clinical markers of ischemic injury, such as time from collapse to CPR, time from collapse to ROSC, serum lactate, intensity of vasopressor therapy, and (3) biomarkers of inflammation (C-reactive protein, procalcitonin). The results of the correlation analyses with categories 1 and 2 are available in the Supplementary Information (see Supplementary Table S2). The composition of monocyte subpopulations did not correlate with 30-day mortality or cardiac arrest specific scoring systems. However, the time to ROSC was significantly associated with an expansion of the intermediate subset at 24 h following CPR (*r*_*s*_: 0.485; *p*: 0.026). Patients with early proof of inflammation, as evidenced by elevated levels of C-reactive protein immediately after hospitalization, had a significantly higher proportion of intermediate monocytes (*r*_*s*_: 0.721; *p*: 0.000) and a lower proportion of classical monocytes (*r*_*s*_: − 0.690; *p*: 0.001) at admission.

### Monocyte CD14 surface expression

**Figure 2:**
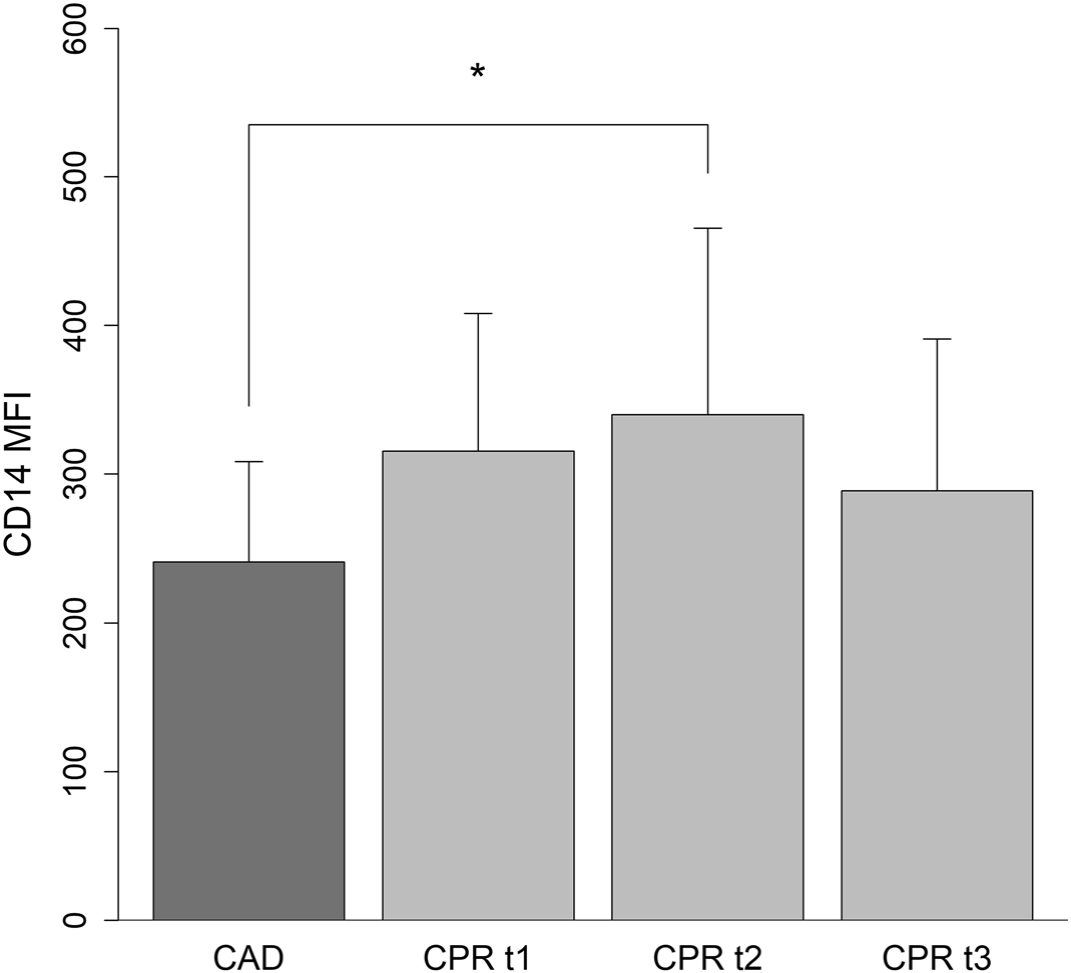
Monocyte CD14 surface expression is increased in patients after cardiac arrest. Shown is the surface expression of CD14 from resuscitated patients’ circulating monocytes during the first 12 h (CPR t1: n = 22), after 24 h (CPR t2: n = 21), and 48 h (CPR t3: n = 16) following cardiac arrest, and from the control group (CAD: n = 19). Statistical hypothesis testing was performed by one-way analysis of variances and post-hoc analysis with all-pairwise comparisons using the Dunn–Bonferroni approach (*: *p* ≤ 0.05). A significant upregulation of CD14 was already observed within the first 12 h (*p* = 0.05) and after 24 h following ROSC (*p* = 0.006), when multiple comparisons between the mean of each column in the CPR group and the mean of the control group were performed using the Dunnett’s approach.

Monocyte PRR signaling pathways, such as TLR2 and TLR4, have been shown to be upregulated during early PCAS^5^. We hypothesized that CD14, the coreceptor of TLR4, would also be upregulated during PCAS and that the level of expression of CD14 might be useful as a biomarker for disease severity. Monocyte CD14 expression levels peaked 24 h post ROSC. Compared to the control group, CD14 expression was significantly higher at this time (CAD 241.11 ± 67.46; CPR t1 315.55 ± 92.46; CPR t2 340.10 ± 125.29; CPR t3 288.81 ± 102.14 (MFI)) (Fig. 2). A correlation between the level of CD14 expression and patient outcomes could not be demonstrated. However, the extent to which CD14 expression was upregulated at 24 h following cardiac arrest was significantly correlated with the time to ROSC (*r*_*s*_: 0.650; *p*: 0.001) (see Supplementary Table S3).

### Monocyte HLA-DR surface expression

**Figure 3:**
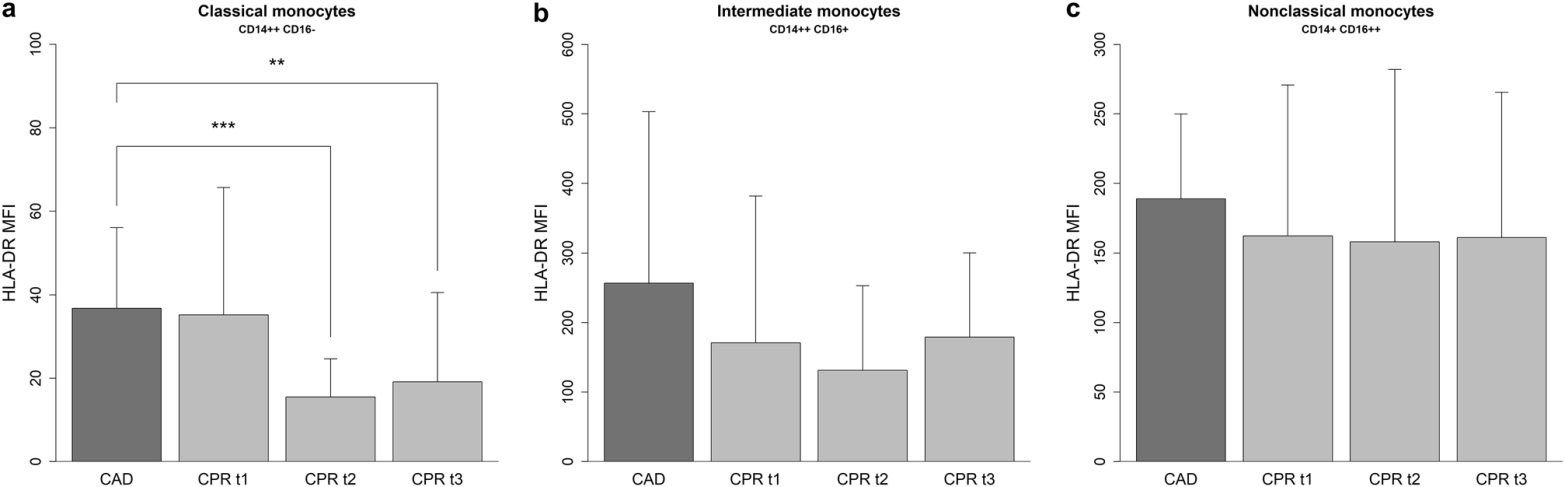
HLA-DR expression on monocyte subpopulations in resuscitated patients and the control group. HLA-DR surface expression levels on monocyte subpopulations of patients from the CPR group in the first 12 h (CPR t1: n = 22), after 24 h (CPR t2: n = 21), and 48 h (CPR t3: n = 16) following cardiac arrest, as well as of patients from the control group (CAD: n = 19) are shown. Statistical hypothesis testing was performed using the Kruskal–Wallis test and post-hoc analysis with all-pairwise comparisons using the Dunn–Bonferroni approach (**: *p* ≤ 0.01; ***: *p* ≤ 0.001).

**Figure 4:**
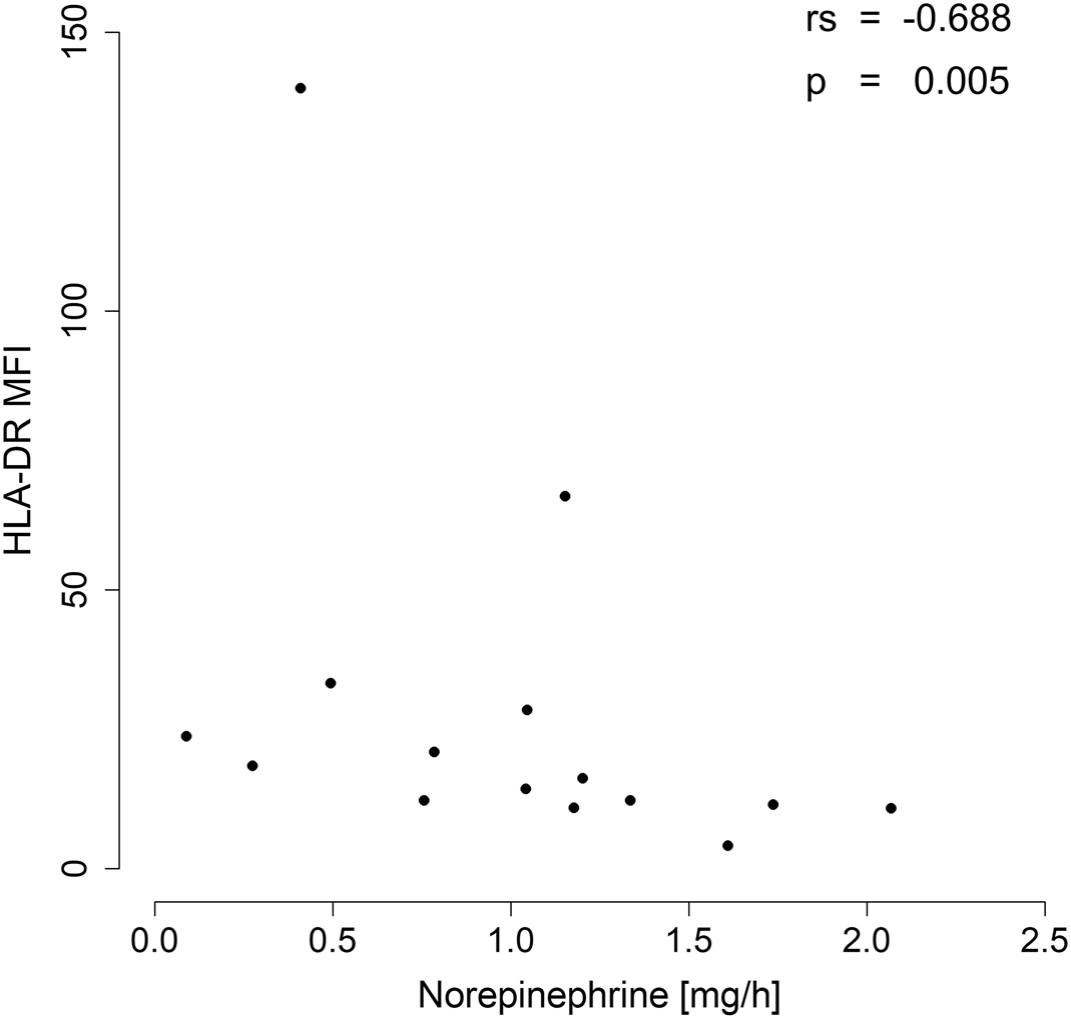
Correlation of monocyte HLA-DR surface expression and dosage of norepinephrine therapy. There was a statistically significant negative correlation between monocyte HLA-DR surface expression and dosage of norepinephrine [mg/h] to maintain a mean arterial blood pressure ≥ 80 mmHg after 48 h following cardiac arrest (CPR t3: *r*_*s*_ = − 0.688; *p* = 0.005).

Downregulation of mHLA-DR is a key feature of monocyte deactivation and represents a diagnostic indicator of immunoparalysis in critically ill patients^12,13^. Compared to the control group, the expression of HLA-DR significantly decreased in resuscitated patients (see Supplementary Fig. S4). Subpopulation analyses revealed that the overall decrease in mHLA-DR expression was the result of downregulation on classical monocytes. Intermediate and nonclassical monocytes, by contrast, showed a similar mean expression of HLA-DR compared to the control group (Fig. 3). However, a subanalysis within the group of resuscitated patients indicated an inhomogeneous expression pattern of HLA-DR on intermediate and nonclassical monocytes depending on the severity of PCAS: First, there was a moderate to strong negative correlation between the likelihood of a poor prognosis, as assessed by cardiac arrest-specific prognostic scores (CAHP, OHCA), and the level of HLA-DR expression on intermediate and nonclassical monocytes. Second, nonsurvivors showed a significantly lower HLA-DR expression on intermediate and nonclassical monocytes in the first 12 h after ROSC, with a trend toward lower values among nonclassical monocytes at 24 h and 48 h post ROSC. Third, patients with an unfavorable neurologic outcome had significantly less expression of HLA-DR on nonclassical monocytes at 24 h and 48 h after ROSC (see Supplementary Table S3). It should be noted that the variance of HLA-DR expression on nonclassical monocytes was considerable in some patients, as this cell population showed a significant decline after cardiopulmonary resuscitation. In addition, total monocyte HLA-DR expression and HLA-DR expression on classical monocytes showed a significant negative correlation with the mean dose of norepinephrine administered for the treatment of post-cardiac arrest shock at 24 h (*r*_*s*_: − 0.474; *p*: 0.030) and 48 h (*r*_*s*_: − 0.688; *p*: 0.005) after ROSC (Fig. 4).

## Discussion

The aim of this study was to further characterize the monocytic inflammatory response during PCAS by analyzing changes in the composition of monocyte subsets and the expression of mHLA-DR and CD14. It was investigated whether these parameters could serve as potential biomarkers for the severity of PCAS and could represent potential future targets for individualized therapy. Our main findings were (1) a significant decrease in the proportion of nonclassical monocytes during early PCAS, (2) a significant upregulation of CD14, which correlated with the time to ROSC, (3) a significant decrease in the expression of mHLA-DR, which was the result of downregulation on classical monocytes and which significantly correlated with the extent of post-cardiac arrest shock, and (4) an inhomogeneous expression of HLA-DR on intermediate and nonclassical monocytes within the CPR group, with patients having a worse prognosis or outcome showing lower HLA-DR levels on these cells.

Our finding of a relative decrease in the number of nonclassical monocytes during the early phase of PCAS was relatively unexpected, since an expansion of this subpopulation has already been described in a variety of systemic inflammatory disorders in noncritically ill patients^14^. However, in accordance with our findings, a similar decrease in nonclassical monocytes has been described in other critically ill patients, such as those with acute liver failure^15^ and sepsis^16^.

From a pathophysiological point of view, systemic endotheliitis is a key feature of PCAS^17,18^. Nonclassical monocytes have been shown to interact with the damaged endothelium in an LFA-1- and CX3CR1-dependent manner^19^. Upregulation of the LFA-1 ligand ICAM-1 has been shown to enhance the interaction between monocytes and endothelial cells in PCAS^20^. The substantial decrease in circulating nonclassical monocytes during PCAS could thus be explained by a larger number of cells patrolling the damaged endothelium. A part of this subpopulation might exit the bloodstream and subsequently redistribute into the injured tissue^11,19^.

Recently, three studies examined the kinetics of monocyte subpopulation distribution during systemic inflammation in the context of human endotoxemia^21–23^, which is a common phenomenon in PCAS^2^. Endotoxin exposure led to profound monocytopenia within 2 h. Subsequently, classical monocytes recovered first (3–6 h), followed by intermediate monocytes (6–8 h) and nonclassical monocytes (24 h). Following systemic inflammation, classical monocytes are rapidly mobilized from the bone marrow and transition over intermediate monocytes to nonclassical monocytes^21^. Considering the developmental relationship of monocyte subpopulations, it could be speculated that, under the assumption of accelerated emergency monopoiesis, mobilization of classical monocytes and maturation to intermediate monocytes had already occurred in the early phase of this study, while the number of nonclassical monocytes was still reduced. Accordingly, restoration of the nonclassical subset in the later phase of this trial could possibly be attributed to de novo maturation from intermediate monocytes.

Alternatively, both an increased cell death rate following bacterial challenge^24^ and glucocorticoid-induced monocytopenia^25^ could possibly explain the relative decrease in nonclassical monocytes after cardiac arrest. Recently, Krychtiuk et al. demonstrated that an expansion of the subset of intermediate monocytes and a decrease of the subset of classical monocytes during the recovery phase of PCAS (> 72 h following cardiac arrest) were associated with poor outcome^26^. The present study focused on the early and intermediate phases of PCAS and therefore provides additional insights into monocyte subset kinetics by revealing the almost complete decline in nonclassical monocytes during the early phase of PCAS.

Circulating leucocytes initiate an inflammatory response to tissue injury by activating PRRs, such as TLRs^4^. CD14, as a coreceptor of TLR4, can be activated by a variety of DAMPs and PAMPs^27^. In line with the hypothesis that CD14 might be involved in systemic inflammation after cardiac arrest, we now observed progressive upregulation of CD14 within 24 h following ROSC. On day 2 after hospital admission, we already observed a decline of CD14. This finding agrees with similar observations in septic patients^28^ and a recently published trial investigating the innate immune response following cardiac arrest^29^. Prolonged stimulation (24–44 h) of monocytes with PAMPs, such as endotoxin, leads to an upregulation of CD14 in vitro^30^. High levels of both endotoxin^2^ and DAMPs^31^ have been reported within the first 24 h of PCAS, which could thus lead to upregulation of CD14. The subsequent decline of these mediators could explain the decrease in CD14 expression after day 2 following ROSC. Furthermore, the expression profile of CD14 is consistent with the mRNA expression profile of TLR signaling pathways during PCAS, as previously demonstrated by our group^5^. Our observation of a significant correlation between the level of CD14 expression and the duration from collapse to ROSC supports a possible relationship between the extent of ischemia–reperfusion injury following cardiac arrest and subsequent monocyte activation.

This study strengthens the hypothesis that resuscitated patients develop early immunoparalysis, which can be characterized by downregulation of mHLA-DR expression, as previously demonstrated^32–34^. However, this is the first study to show that HLA-DR expression is altered primarily among classical monocytes. This observation contrasts with results from a previously published trial, which showed that mHLA-DR expression decreased on both CD14^++^ and CD14^+^ monocytes after cardiac arrest^32^. This discrepancy can possibly be attributed to the difference in the classification of monocyte subsets, since in the latter study, a division into only two monocyte subsets was performed. Although HLA-DR expression on intermediate and nonclassical monocytes did not change on average in the present study, an inhomogeneous expression pattern on these cells was observed regarding the disease severity of PCAS. Interestingly, an unfavorable prognosis at the time of admission, as assessed by the CAHP and the OHCA score, was associated with significantly less HLA-DR expression on intermediate and nonclassical monocytes. Nonsurvivors and patients with an unfavorable neurological outcome had lower HLA-DR levels, although this effect was not consistent at all time points and on both subpopulations.

Downregulation of HLA-DR is mainly mediated by glucocorticoids^35^ and interleukin 10 (IL-10)^36^. Both elevated cortisol levels^37^ and excessive secretion of IL-10^2^ have been reported in patients after cardiac arrest. While glucocorticoids cause downregulation of HLA-DR on both CD14^++^ and CD14^+^ monocytes, IL-10 primarily leads to downregulation of HLA-DR on CD14^++^ monocytes^36^. The inhomogeneous expression pattern of HLA-DR on intermediate and nonclassical monocytes among the CPR group and its correlation with disease severity could possibly be explained by increased glucocorticoid levels, because nonsurvivors, especially patients with hypoxic brain injury, show significantly higher cortisol levels^38^.

In the present study, a significant correlation between downregulation of mHLA-DR and the dose of norepinephrine administered to treat post-cardiac arrest shock was observed. Catecholamines have been shown to trigger the release of IL-10 which is known to downregulate HLA-DR on monocytes^38,39^. The role of norepinephrine as an intermediate factor linking disease severity to the development of immunosuppression is the subject of current research in the field of sepsis^40^. Since patients after cardiac arrest show markedly elevated levels of endogenous catecholamines^41^ and are treated with high doses of catecholamines in the context of post-cardiac arrest shock, further investigations should be sought regarding a possible role of norepinephrine in the context of monocyte deactivation during PCAS.

## Study limitations

Due to its unique pathophysiology and the heterogeneity of the study population, the choice of an appropriate reference group in cardiac arrest research remains challenging. Patients with CAD were enrolled in the reference group to control for the confounder of ischemic heart disease, which is known for monocyte activation and which was the major cause of cardiac arrest in our study cohort. Aside from intensive care, both cohorts received similar interventional and pharmacological treatments. Since the control group was not exposed to targeted temperature management at 33 °C, this factor may have contributed as a confounder, as cooling potentially contributes to attenuation of inflammatory damage after ischemia–reperfusion injury^42^. However, cooling does not seem to affect HLA-DR expression after cardiac arrest, as previously described^32^. To overcome the dilemma of finding a suitable reference group in cardiac arrest research, serial measurements were performed in the CPR group as an internal control to evaluate the dynamics of the parameters investigated individually in each patient over time. Since this study focused on circulating monocytes, the inflammatory profile of monocytes redistributing to the inflamed tissue remains beyond the scope of this study. Due to the inbuilt limitations of observational studies, causation between our findings and the development of PCAS cannot be concluded due to possible confounders. Furthermore, the study was conducted at a single center with a rather small sample size of 32 patients admitted to intensive care after CPR.

## Conclusions

It is believed that circulating monocytes play an important role in initiating and perpetuating systemic inflammation during PCAS. Our descriptive findings in 32 patients suggest that PCAS can be characterized by profound dysregulation of monocyte immune homeostasis at an early stage. Regarding the composition of circulating monocyte subpopulations, we observed a significant decline in the nonclassical subset, which is well known for its function as a “housekeeper” of the endothelium. On the one hand, we observed monocyte activation, demonstrated by an upregulation of CD14; on the other hand we simultaneously detected monocyte deactivation, characterized by downregulation of HLA-DR, which was significantly correlated with disease severity. While the latter immunosuppressive mechanism may be beneficial in limiting secondary damage caused by excessive inflammation, it may render the organism more susceptible to secondary infectious complications. At the present time, apart from targeted temperature management, no specific therapy for PCAS has been developed. Immunomodulatory therapy represents one possible approach to close this therapeutic gap. It can be speculated that patients with PCAS, comparable to patients with sepsis^43^, undergo different immunological states during different phases of the disease. Since a variety of antiinflammatory therapies have failed to provide benefit in patients with sepsis, substances are currently being investigated to attenuate sepsis-induced immunosuppression^44^. A similar therapeutic approach would also be conceivable in patients with PCAS. Therefore, the current data should encourage further immunophenotyping during the time course of PCAS to potentially provide a more individualized therapy approach to this patient population by immunomodulation. To what extent the present findings determine the course of PCAS and in how far immunomodulatory therapies could be beneficial for these patients needs further clarification.

## Methods

### Patient recruitment

This study was approved by the ethics committee of the University Medical Center Freiburg (approval number 328/09) and conforms to the tenets of the Declaration of Helsinki. During 08/2011 and 08/2012, patients who had undergone CPR and were admitted to the internal intensive care unit at the University Medical Center Freiburg, Germany, were prospectively enrolled in this study, as previously described^5^. A total of 33 patients after CPR were prospectively enrolled during the study period. All patients received standardized PCAS treatment, including targeted temperature management at 33 °C^1^. Informed consent was obtained retrospectively from patients who survived to hospital discharge with good neurological outcome. The patients’ next of kin were informed about the study details. Twenty patients with both stable and unstable coronary artery disease (CAD), but without acute myocardial infarction, who were treated as inpatients in our cardiology department, were enrolled as control subjects during the study period. Written informed consent was obtained from all patients in the control group. One case and one control subject were retrospectively excluded due to violation of the exclusion criteria, which was not evident at the time of enrollment. The trial was registered in the German Clinical Trials Register (DRKS00009684).

### Inclusion and exclusion criteria

Patients who underwent CPR ≥ 5 min, aged 18 years and older, were prospectively enrolled. Patients with traumatic cardiac arrest, patients with pre-existing infectious or inflammatory diseases, patients who were taking immunosuppressive medication, and patients with apparent multiple organ dysfunction prior to CPR were excluded.

### Sample collection

Blood samples were collected from an arterial line into a tripotassium ethylenediaminetetraacetic acid (EDTA) anticoagulated tube (Sarstedt, Nürnbrecht, Germany) within 12 h, after 24 h and 48 h following admission. One blood specimen was collected by sterile venipuncture from control subjects. Samples were drawn slowly and immediately processed. If a sample collection could not be performed at a given time point in the CPR group (death of the patient, transfer of the patient, or logistical reasons) the patient remained enrolled in the study, and the remaining blood samples were collected according to the study protocol. A total of 22 blood samples were analyzed within the first 12 h after admission in the CPR group, 21 blood samples after 24 h, and 16 blood samples after 48 h.

### Flow cytometric analyses

**Figure 5:**
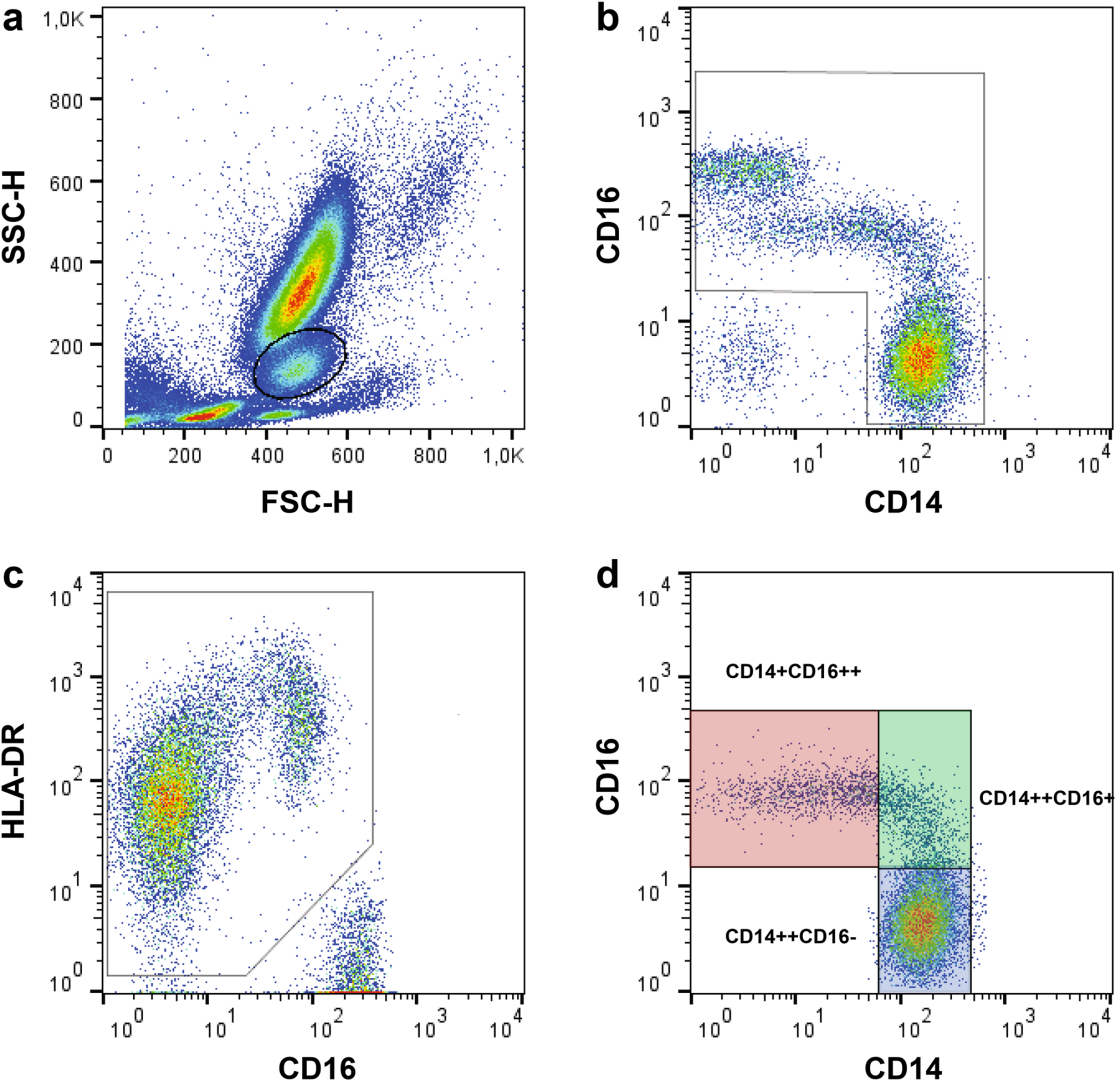
Positive inclusion gating strategy of monocyte subset populations. Monocyte subpopulations were identified using a positive inclusion gating strategy^15^. Monocytes were first visualized on a forward and side light scattered plot, and an ample gate was drawn around the monocyte cloud to exclude most neutrophils, lymphocytes, and cell debris (**a**). Monocytes were identified in the daughter population on a CD14/CD16 dot plot (**b**). Subsequently, the remaining cell population was visualized on a CD16/HLA-DR dot plot, allowing the CD16^+^/HLA-DR^−^ nonmonocytic cells to be excluded (**c**). Monocyte subpopulations were identified via a rectangular gating strategy (**d**).

EDTA anticoagulated blood (100 µl) was diluted with 200 µl Dulbecco’s phosphate-buffered saline solution (DPBS) without CaCl_2_ and MgCl_2_ (Life Technologies, Carlsbad, CA, USA), which was supplemented with 1% sterile-filtered bovine serum albumin solution (Sigma-Aldrich, St. Louis, USA). Blood samples were processed in duplicate for triple staining with 10 µl phycoerythrin (PE) anti-human CD14 antibody (Beckman-Coulter, Krefeld, Germany), 10 µl fluorescein isothiocyanate (FITC) anti-human CD16 antibody (Beckman-Coulter, Krefeld, Germany), and 20 µl allophycocyanin (APC) anti-human HLA-DR antibody (Abcam plc, Cambridge, UK). Another blood sample was stained with 10 µl FITC anti-mouse IgG1 antibody (Beckman-Coulter, Krefeld, Germany), 10 µl PE anti-mouse IgG1 antibody (Beckman Coulter, Krefeld, Germany), and 10 µl APC anti-mouse IgG1 antibody (BD Biosciences, San Jose, USA), which served as a negative control. After 30 min of incubation in the dark, the cell suspension was washed with 1 ml DPBS by centrifugation at 400 × g for 3 min. The supernatant was discarded, 2 ml lysis buffer (BD FACS™ Lysing Solution, BD Biosciences, San Jose, USA) was added, and the solution was thoroughly vortexed. After 5 min of incubation in the dark, the samples were centrifuged at 400 × g for 3 min. The supernatant was discarded, and the samples were washed twice with DPBS. Stained cells were resuspended in 300 µl fixation buffer (BD CellFIX™, BD Biosciences, San Jose, USA) and immediately analyzed by flow cytometry (BD FACSCalibur™, BD Biosciences, San Jose, USA). Samples were acquired using CellQuest™ software (BD Biosciences, San Jose, USA). The results were generated from the FCS3 data file using FlowJo V10 software (FlowJo LLC, Ashland, USA). Monocyte subpopulations were identified using a positive inclusion gating strategy based on their expression of CD14, CD16, and HLA-DR (Fig. 5)^15^. The absolute monocyte count was calculated from the white blood cell count, which was determined by our central laboratory, based on the relative fraction of monocytes, as assessed by our flow cytometry data. The median fluorescence intensity (MFI) of CD14 and HLA-DR was recorded for each monocyte subset.

### Statistics

Statistical analysis was performed using SPSS 21 (IBM, Armonk, USA) and GraphPad Prism 9 (GraphPad Software, San Diego, USA). Normal distribution was verified by the Shapiro–Wilk test, visualization of the respective histograms, and calculation of the Z-score of skewness and kurtosis. The assumption of homogeneity of variances was assessed by the nonparametric Levene test. Fisher’s exact test was used to compare categorical variables. Normally distributed unpaired data consisting of multiple groups were analyzed with one-way analysis of variances and post-hoc analysis with all-pairwise comparison. Nonnormally distributed data consisting of multiple groups were analyzed with the Kruskal–Wallis test and a post-hoc analysis using the Dunn–Bonferroni approach. As indicated, in a few exceptional analyses, a multiple comparison between the mean of each timepoint in the CPR group was compared to the mean of the control group in addition to an all-pairwise comparison. Correlation analyses were performed using Spearman’s rank correlation. Statistical significance was defined as a two-tailed *p* value ≤ 0.05. Continuous variables are reported as the mean value ± standard deviation.

## Supplementary Information

Supplementary Information.

## Data Availability

The datasets generated and/or analyzed during the current study are available from the corresponding author upon reasonable request.

## Abbreviations

APACHE II score: Acute Physiology and Chronic Health Evaluation Score II
APC: Allophycocyanin
CAD: Coronary artery disease
CAHP score: Cardiac Arrest Hospital Prognosis Score
CPC: Cerebral performance category
CPR: Cardiopulmonary resuscitation
DAMPs: Danger-associated molecular patterns
DPBS: Dulbecco’s phosphate-buffered saline
EDTA: Ethylenediaminetetraacetic acid
FITC: Fluorescein isothiocyanate
HLA-DR: Human leucocyte antigen-DR
IL-10: Interleukin 10
MFI: Median fluorescence intensity
mHLA-DR: Monocyte HLA-DR
OHCA score: Out-of-Hospital Cardiac Arrest Score
PAMPs: Pathogen-associated molecular patterns
PCAS: Post-cardiac arrest syndrome
PE: Phycoerythrin
PRR: Pattern recognition receptor
ROSC: Return of spontaneous circulation
SAPS II score: Simplified Acute Physiology Score II
TLR: Toll-like receptor
