# Supplementary material for: Monocyte subset distribution and surface expression of HLA-DR and CD14 in patients after cardiopulmonary resuscitation
Source: Sci Rep. 2021 Jun 11;11:12403. doi: 10.1038/s41598-021-91948-z (PMC8196031; doi:10.1038/s41598-021-91948-z)
Supplement: Supplementary file 1 — Supplementary Information. [file 41598_2021_91948_MOESM1_ESM.pdf]

Monocyte subset distribution and surface expression of HLA-DR and CD14 in patients after cardiopulmonary resuscitation

Alexander Asmussen[1*], MD, Hans-Jörg Busch[2], MD, Thomas Helbing[3], MD, Xavier Bemtgen[1], MD, Christian Smolka[1], PhD, Christoph Bode[1], MD, Katrin Fink[2#], MD, Sebastian Grundmann[1#], MD, PhD

[1] Department of Cardiology and Angiology I, University Heart Center Freiburg – Bad Krozingen, University Medical Center Freiburg, Faculty of Medicine, University of Freiburg
Hugstetter Str. 55, 79106 Freiburg im Breisgau, Germany

[2] Department of Emergency Medicine, University Medical Center Freiburg, Faculty of Medicine, University of Freiburg
Sir-Hans-A.-Krebs-Straße, 79106 Freiburg im Breisgau, Germany

[3] Department of Cardiology, Heart Center Oldenburg, University of Oldenburg
Rahel-Straus-Str. 10, 26133 Oldenburg, Germany

* Corresponding author; [#] These two authors contributed equally to this work

Supplementary Figure S1: Absolute monocyte subset count in resuscitated patients and the control group.

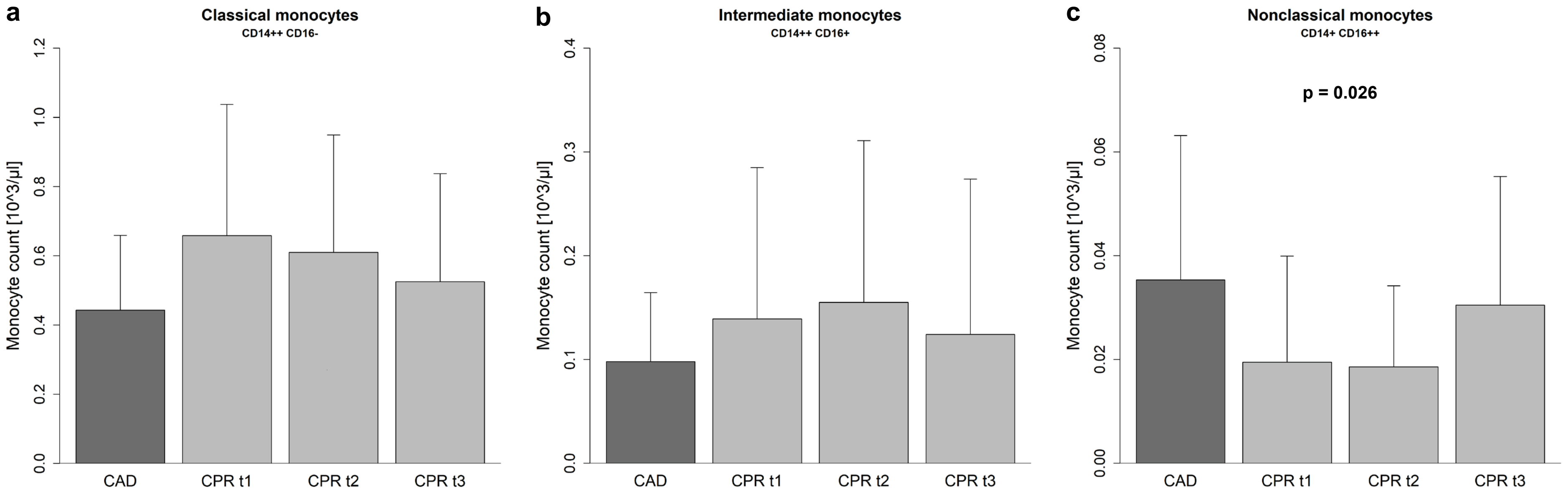

Shown is the absolute count of monocyte subsets from the CPR group in the first 12 h (CPR t1: n = 22), after 24 h (CPR t2: n = 21) and 48 h (CPR t3: n = 16) following ROSC, and the control group (CAD: n = 19). Statistical hypothesis testing was performed using the Kruskal-Wallis test. The Kruskal-Wallis test indicated a significant difference in the number of nonclassical monocytes between the four groups (p = 0.026). Post-hoc analysis with all-pairwise comparisons using the Dunn-Bonferroni approach showed only a trend to lower numbers of nonclassical monocytes in the first 12 h (CPR t1 vs CAD group, p = 0.068) and after 24 h following ROSC (CPR t2 vs CAD group, p = 0.157). However, when post-hoc multiple comparisons with the Dunn’s approach were applied, where the means of every column in the CPR group were compared only to the control group, we did observe a significant lower count of nonclassical monocytes in the first 12 h (CPR t1 vs CAD group, p = 0.034) and a trend to lower numbers of nonclassical monocytes at 24 h following ROSC (CPR t2 vs CAD group, p = 0.078).

Supplementary Table S2: Spearman's rank correlation between monocyte subset composition and clinical characteristics

			Time to ROSC	Time to CPR	CPC (3-5)	30-day mortality	SOFA score	SAPS II score	APACHE II score	CAHP score	OHCA score	Nor-epinephrine [mg/h]
**$CD14^{++}CD16^{-}$ [%]**	T1	r_s:	-0.157	0.013	-0.040	-0.050	-0.293	-0.067	0.041	0.116	0.122	0.174
		p:	0.509	0.957	0.836	0.829	0.186	0.767	0.856	0.618	0.599	0.438
	T2	r_s:	-0.414	0.054	0.295	-0.017	0.178	0.079	**0.434**	-0.208	-0.282	0.040
		p:	0.062	0.818	0.194	0.943	0.440	0.734	**0.049**	0.380	0.228	0.862
	T3	r_s:	0.065	-0.091	-0.280	-0.451	0.378	-0.177	0.342	-0.042	0.044	0.407
		p:	0.812	0.738	0.293	0.080	0.165	0.513	0.194	0.887	0.881	0.132
**$CD14^{++}CD16^{-}$ [10^3/µl]**	T1	r_s:	0.032	-0.103	-0.080	**-0.467**	0.295	-0.364	-0.170	-0.083	-0.306	0.159
		p:	0.892	0.666	0.730	**0.033**	0.183	0.096	0.451	0.720	0.178	0.480
	T2	r_s:	0.154	-0.048	0.018	-0.234	-0.320	-0.025	0.245	0.057	-0.104	-0.087
		p:	0.505	0.838	0.937	0.308	0.157	0.915	0.285	0.811	0.663	0.708
	T3	r_s:	-0.480	0.057	0.308	0.096	0.372	0.320	0.243	-0.051	-0.082	0.239
		p:	0.060	0.834	0.246	0.725	0.172	0.228	0.365	0.864	0.781	0.390
**$CD14^{++}CD16^{+}$ [%]**	T1	r_s:	0.140	-0.076	0.020	0.067	0.336	0.032	-0.072	-0.097	-0.117	-0.202
		p:	0.555	0.749	0.931	0.774	0.126	0.888	0.749	0.674	0.613	0.366
	T2	r_s:	**0.485**	-0.049	-0.305	-0.008	-0.221	-0.093	**-0.449**	0.240	0.279	-0.064
		p:	**0.026**	0.832	0.179	0.971	0.335	0.689	**0.041**	0.308	0.234	0.784
	T3	r_s:	-0.016	-0.222	0.028	0.232	-0.247	0.244	-0.452	-0.090	-0.155	-0.268
		p:	0.953	0.408	0.918	0.387	0.374	0.361	0.078	0.759	0.597	0.334
**$CD14^{++}CD16^{+}$ [10^3/µl]**	T1	r_s:	0.079	0.048	0.140	-0.067	0.416	-0.122	-0.123	-0.012	-0.128	-0.223
		p:	0.740	0.840	0.544	0.774	0.054	0.589	0.584	0.960	0.582	0.319
	T2	r_s:	**0.598**	-0.066	-0.203	-0.050	-0.260	-0.207	-0.245	0.301	0.324	-0.104
		p:	**0.004**	0.778	0.377	0.829	0.255	0.367	0.284	0.198	0.164	0.654
	T3	r_s:	-0.341	-0.156	0.336	0.342	0.259	0.343	-0.179	-0.095	-0.175	0.007
		p:	0.196	0.564	0.203	0.195	0.352	0.193	0.508	0.748	0.550	0.980
**$CD14^{+}CD16^{++}$ [%]**	T1	r_s:	-0.135	0.388	0.360	0.100	0.212	0.099	0.174	-0.077	-0.019	-0.039
		p:	0.570	0.091	0.108	0.666	0.344	0.662	0.440	0.741	0.935	0.863
	T2	r_s:	-0.287	0.194	-0.074	0.167	0.015	-0.140	-0.233	0.003	-0.044	0.221
		p:	0.207	0.399	0.750	0.470	0.950	0.546	0.310	0.990	0.855	0.336
	T3	r_s:	-0.043	0.237	0.252	0.396	**-0.557**	0.230	0.113	0.226	0.075	-0.250
		p:	0.875	0.377	0.346	0.129	**0.031**	0.392	0.677	0.436	0.798	0.369
**$CD14^{+}CD16^{++}$ [10^3/µl]**	T1	r_s:	-0.152	0.258	0.340	-0.067	**0.487**	-0.251	-0.083	-0.068	-0.217	-0.032
		p:	0.521	0.271	0.131	0.774	**0.021**	0.260	0.715	0.771	0.346	0.889
	T2	r_s:	-0.051	0.066	-0.111	-0.050	-0.306	-0.167	-0.090	0.015	-0.096	-0.013
		p:	0.827	0.778	0.633	0.829	0.177	0.470	0.697	0.950	0.686	0.955
	T3	r_s:	-0.359	**0.508**	0.476	0.396	-0.306	0.449	0.252	0.305	0.246	-0.018
		p:	0.172	**0.044**	0.062	0.129	0.268	0.081	0.347	0.288	0.397	0.950

Correlation analyses of clinical characteristics in the CPR group and monocyte subset composition during the first 12 h (CPR t1; n = 22), after 24 h (CPR t2; n = 21), and 48 h (CPR t3; n = 16) following ROSC are shown. One patient was lost to follow-up after study enrollment. Statistical hypothesis testing was performed using Spearman's rank correlation, the result of which is reported as Spearman's rho (r_s) and the *p*-values listed above. CAHP and OHCA score were only calculated in patients after out-of-hospital cardiac arrest.
Time to ROSC, time from collapse to return of spontaneous circulation; Time to CPR, time from collapse to cardiopulmonary resuscitation; SOFA score, Sequential Organ Failure Assessment score; SAPS II score, Simplified Acute Physiology Score II, APACHE II score, Acute Physiology and Chronic Health Evaluation II score; CAHP score, Cardiac Arrest Hospital Prognosis score; OHCA score, Out-of-Hospital Cardiac Arrest score; Norepinephrine [mg/h], dose of norepinephrine, which was necessary to maintain a mean arterial blood pressure ≥ 80 mmHg

Supplementary Table S3: Spearman's rank correlation of CD14 and HLA-DR expression with clinical characteristics

			Time to ROSC	Time to CPR	CPC (3-5)	30-day mortality	SOFA score	SAPS II score	APACHE II score	CAHP score	OHCA score	Nor-epinephrine [mg/h]
**CD14 MFI**	T1	r_s:	0.006	0.024	-0.240	-0.217	0.273	-0.264	-0.092	-0.236	-0.204	-0.049
		p:	0.980	0.920	0.294	0.345	0.220	0.236	0.685	0.302	0.376	0.830
	T2	r_s:	**0.650**	-0.243	-0.277	-0.100	-0.012	-0.145	0.016	0.176	0.288	-0.256
		p:	**0.001**	0.289	0.224	0.666	0.959	0.530	0.946	0.458	0.218	0.263
	T3	r_s:	-0.028	0.036	0.196	0.096	0.009	-0.467	0.064	0.099	0.217	-0.300
		p:	0.918	0.896	0.467	0.725	0.973	0.068	0.814	0.737	0.457	0.277
**HLA-DR MFI**	T1	r_s:	-0.247	-0.105	-0.300	-0.367	-0.215	0.150	-0.348	-0.352	-0.241	-0.235
		p:	0.295	0.658	0.186	0.102	0.337	0.505	0.113	0.118	0.293	0.293
	T2	r_s:	0.110	-0.185	-0.222	-0.184	-0.256	**-0.438**	-0.393	-0.192	-0.081	**-0.474**
		p:	0.635	0.423	0.334	0.426	0.263	**0.047**	0.078	0.416	0.736	**0.030**
	T3	r_s:	-0.249	**-0.549**	-0.126	0.027	-0.070	-0.234	**-0.681**	**-0.704**	**-0.751**	**-0.688**
		p:	0.353	**0.028**	0.642	0.920	0.804	0.384	**0.004**	**0.005**	**0.002**	**0.005**
**HLA-DR MFI $CD14^{++}CD16^{-}$**	T1	r_s:	-0.271	-0.083	-0.300	-0.317	-0.260	0.178	-0.280	-0.310	-0.187	-0.181
		p:	0.247	0.728	0.186	0.162	0.242	0.428	0.207	0.171	0.418	0.420
	T2	r_s:	0.097	-0.210	-0.222	-0.200	-0.299	-0.306	-0.250	-0.106	-0.052	**-0.470**
		p:	0.675	0.361	0.334	0.384	0.188	0.177	0.274	0.656	0.829	**0.031**
	T3	r_s:	-0.390	**-0.606**	-0.112	-0.041	0.148	-0.229	**-0.658**	**-0.805**	**-0.807**	**-0.584**
		p:	0.136	**0.013**	0.679	0.880	0.599	0.394	**0.006**	**0.001**	**0.000**	**0.022**
**HLA-DR MFI $CD14^{++}CD16^{+}$**	T1	r_s:	-0.364	-0.307	-0.360	**-0.484**	-0.206	0.117	-0.092	**-0.666**	**-0.557**	0.071
		p:	0.115	0.189	0.108	**0.026**	0.357	0.605	0.685	**0.001**	**0.009**	0.753
	T2	r_s:	-0.311	-0.255	0.092	-0.100	-0.188	-0.263	-0.225	**-0.517**	**-0.547**	-0.404
		p:	0.170	0.265	0.691	0.666	0.414	0.250	0.327	**0.020**	**0.012**	0.069
	T3	r_s:	-0.211	-0.486	-0.238	-0.246	0.318	-0.424	**-0.593**	**-0.726**	**-0.659**	-0.336
		p:	0.434	0.056	0.374	0.358	0.247	0.101	**0.016**	**0.003**	**0.010**	0.221
**HLA-DR MFI $CD14^{+}CD16^{++}$**	T1	r_s:	-0.329	-0.227	-0.320	**-0.500**	-0.108	0.075	-0.078	**-0.579**	**-0.433**	-0.101
		p:	0.157	0.336	0.157	**0.021**	0.633	0.739	0.730	**0.006**	**0.050**	0.656
	T2	r_s:	0.201	**-0.552**	**-0.499**	-0.400	-0.033	-0.190	-0.325	-0.362	-0.230	-0.300
		p:	0.383	**0.009**	**0.021**	0.072	0.889	0.410	0.150	0.116	0.330	0.186
	T3	r_s:	0.219	**-0.667**	**-0.504**	-0.478	0.142	**-0.551**	**-0.534**	**-0.776**	**-0.615**	-0.139
		p:	0.415	**0.005**	**0.046**	0.061	0.615	**0.027**	**0.033**	**0.001**	**0.019**	0.621

Correlation analyses of clinical characteristics in the CPR group and monocyte surface expression of CD14 and HLA-DR during the first 12 h (CPR t1; n = 22), after 24 h (CPR t2; n = 21), and 48 h (CPR t3; n = 16) following ROSC are shown. One patient was lost to follow-up after study enrollment. Statistical hypothesis testing was performed using Spearman's rank correlation, the result of which is reported as Spearman's rho (r_s) and the *p*-values listed above. CAHP and OHCA score were only calculated in patients after out-of-hospital cardiac arrest.

Time to ROSC, time from collapse to return of spontaneous circulation; Time to CPR, time from collapse to cardiopulmonary resuscitation; SOFA score, Sequential Organ Failure Assessment score; SAPS II score, Simplified Acute Physiology Score II, APACHE II score, Acute Physiology and Chronic Health Evaluation II score; CAHP score, Cardiac Arrest Hospital Prognosis score; OHCA score, Out-of-Hospital Cardiac Arrest score; Norepinephrine [mg/h], dose of norepinephrine, which was necessary to maintain a mean arterial blood pressure ≥ 80 mmHg

Supplementary Figure S4: HLA-DR surface expression on circulating monocytes of patients after cardiac arrest and the control group.

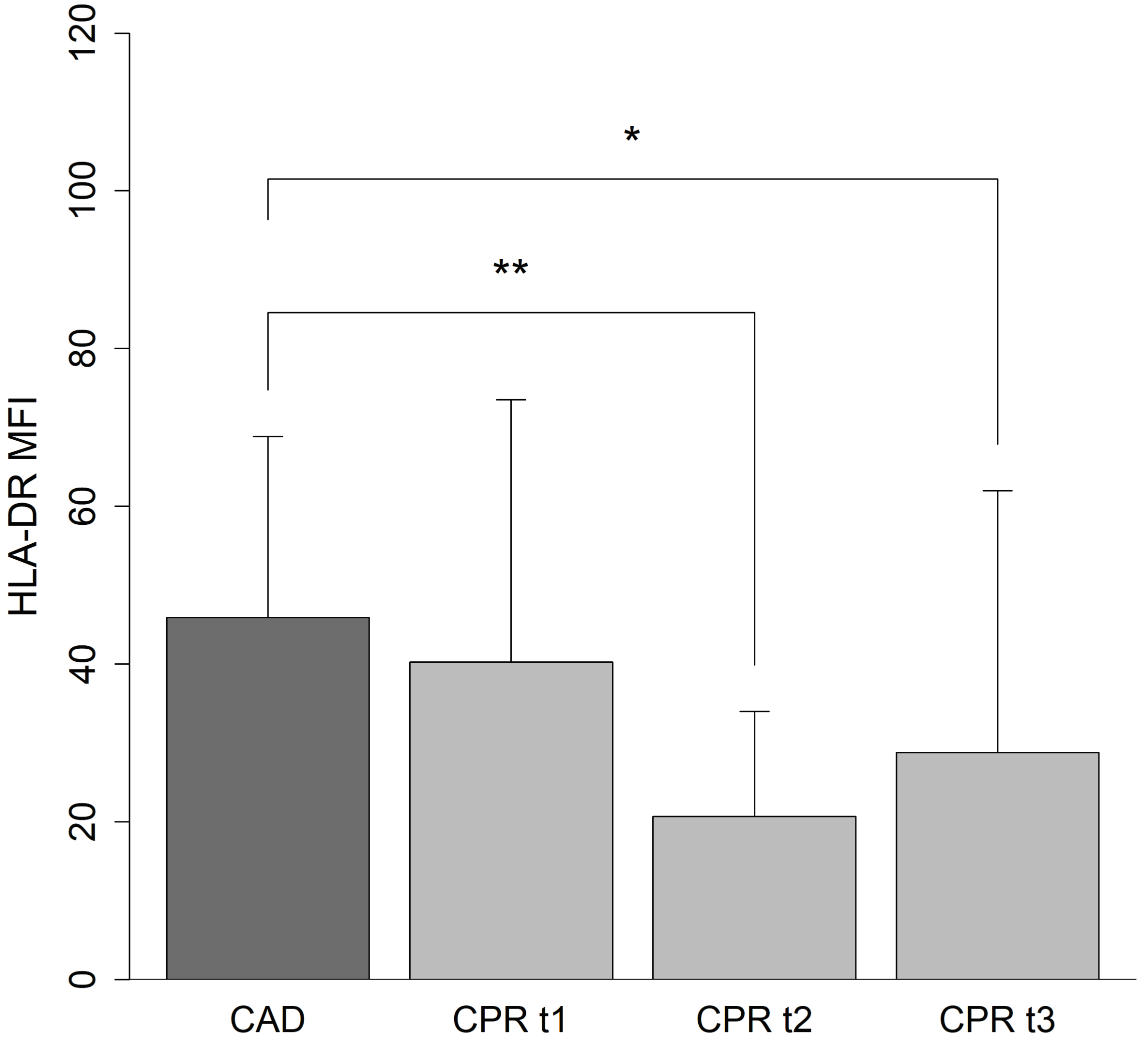

The surface expression of HLA-DR (expressed as median fluorescence intensity) on circulating monocytes of patients during the first 12 h (CPR t1; n = 22), after 24 h (CPR t2; n = 21), and 48 h (CPR t3; n = 16) post ROSC and of patients from the control group (CAD; n = 19) is shown. Statistical hypothesis testing was performed using the Kruskal-Wallis test and post-hoc analysis with all-pairwise comparisons using the Dunn-Bonferroni approach (*: $p \leq 0.05$; **: $p \leq 0.01$).
